# The Glutamate Receptor-Like Protein GLR3.7 Interacts With 14-3-3ω and Participates in Salt Stress Response in *Arabidopsis thaliana*

**DOI:** 10.3389/fpls.2019.01169

**Published:** 2019-09-30

**Authors:** Po-Hsun Wang, Cheng-En Lee, Yi-Sin Lin, Man-Hsuan Lee, Pei-Yuan Chen, Hui-Chun Chang, Ing-Feng Chang

**Affiliations:** ^1^Institute of Plant Biology, National Taiwan University, Taipei, Taiwan; ^2^Department of Life Science, National Taiwan University, Taipei, Taiwan; ^3^Genome and Systems Biology Degree Program, National Taiwan University and Academia Sinica, Taipei, Taiwan

**Keywords:** *Arabidopsis*, glutamate receptor, kinase, phosphorylation, 14-3-3, root, salt, calcium

## Abstract

Ionotropic glutamate receptors (iGluRs) are ligand-gated cation channels that mediate fast excitatory neurotransmission in the mammalian central nervous system. In the model plant *Arabidopsis thaliana*, a family of 20 glutamate receptor-like proteins (GLRs) shares similarities to animal iGluRs in sequence and predicted secondary structure. However, the function of GLRs in plants is little known. In the present study, a serine site (Ser-860) of AtGLR3.7 phosphorylated by a calcium-dependent protein kinase (CDPK) was identified and confirmed by an *in vitro* kinase assay. Using a bimolecular fluorescence complementation and quartz crystal microbalance analyses, the physical interaction between AtGLR3.7 and the 14-3-3ω protein was confirmed. The mutation of Ser-860 to alanine abolished this interaction, indicating that Ser-860 is the 14-3-3ω binding site of AtGLR3.7. Compared with wild type, seed germination of the *glr3.7-2* mutant was more sensitive to salt stress. However, the primary root growth of GLR3.7-S860A overexpression lines was less sensitive to salt stress than that of the wild-type line. In addition, the increase of cytosolic calcium ion concentration by salt stress was significantly lower in the *glr3.7-2* mutant line than in the wild-type line. Moreover, association of 14-3-3 proteins to microsomal fractions was less in GLR3.7-S860A overexpression lines than in GLR3.7 overexpression line under 150 mM NaCl salt stress condition. Overall, our results indicated that *GLR3.7* is involved in salt stress response in *A. thaliana* by affecting calcium signaling.

## Introduction

Abiotic stresses, such as flooding, drought, cold, salt, and heat stresses, limit plant growth and reduce crop yield. Among these, salt stress affects plants the most because of both osmotic and ion toxicity effects. Plants respond to the osmotic effect of salt stress by accumulating compatible solutes to adjust osmotic potential. Using a salt exclusion strategy, plants reduce salt in the cytosol through sodium proton (Na^+^) antiporters. For example, salt overly sensitive 1 (SOS1) is regulated by SOS2 and SOS3 and transports Na^+^ out of plant cells (Zhu, 2001). In addition, plants respond to salt stress through specific signal transduction pathways. Abscisic acid (ABA)–related gene expression is up-regulated and controls salt stress responses in plants under salt stress (Shinozaki and Yamaguchi-Shinozaki, 2007; Kudla et al., 2010).

The *Arabidopsis* genome contains about 57 putative cation-selective channels (Véry and Sentenac, 2002). Among calcium (Ca^2+^) channels, there are Ca^2+^ influx channels that are thermodynamically passive and Ca^2+^ efflux channels that are thermodynamically active. Cyclic nucleotide-gated channels, glutamate receptor-like channels (GLRs), and two-pore channels have been reported for Ca^2+^ influx in previous studies (Frietsch et al., 2007; Tapken and Hollmann, 2008), while Ca^2+^ exchanger antiporters and P-type ATPase pumps have been reported for Ca^2+^ efflux (Pittman et al., 2002; Shigaki et al., 2006). To maintain Ca^2+^ concentration in the cytoplasm, the functioning of influx and efflux channels is in a dynamic equilibrium.

In mammals, GLRs play essential roles in cell-to-cell communication in the nervous system. In *Arabidopsis thaliana*, 20 GLRs (AtGLRs) share substantial similarity in protein sequence and predicted secondary structure with animal ionotropic glutamate receptor (iGluR) subunits (Lam et al., 1998). Sequence similarities are shared among all known iGluR subunits, including the α-amino-3-hydroxy-5-methyl-4-isoxazolepropionic acid receptor (AMPA), kainate receptor, *N*-methyl-d-aspartate (NMDA), and δ-receptors, suggesting that they also share a similar architecture. In plant cells, exogenous amino acids trigger a large, transient increase in cytosolic Ca^2+^ concentration (Dennison and Spalding, 2000; Stephens et al., 2008). In this case, the function of plant GLRs is similar to that of iGluRs (Chiu et al., 1999). However, the biological roles of GLR subunits in plants have not been clearly defined.

Ionotropic glutamate receptors are integral membrane proteins composed of four large subunits (homo-tetramers or hetero-tetramers) that form a central ion channel pore. The first evidence of this quaternary structure was provided by a single particle image of recombinant and native AMPA receptors obtained by electron microscopy (Safferling et al., 2001; Tichelaar et al., 2004). *Arabidopsis* GLR channel structures have three transmembrane domains and an ion-pore loop when subunits combine into a functional tetramer (Colquhoun and Sivilotti, 2004) and, similar to iGluRs, present a putative ligand-binding domain formed by the interactions of the S1 and S2 domains.

*Arabidopsis* phenotypes resulting from mutations in or altered expressions of *GLRs* have provided different information. While *AtGLR1.1* is involved in carbon/nitrogen (C/N) sensing (Kang and Turano, 2003), *AtGLR3.2*, *AtGLR3.3* and *AtGLR3.6* mediate leaf to leaf wound signaling (Mousavi et al., 2013); *AtGLR3.6* also affects primary and lateral root development through cell cycle control (Singh et al., 2016; Toyota et al., 2018). Although *AtGLR3.2, AtGLR3.3*, and *AtGLR3.4* affect lateral root development *via* Ca^2+^ signaling in the phloem (Vincill et al., 2013), the overexpression of *AtGLR3.1* affected stomata closing behavior without affecting cytosolic Ca^2+^ (Cho et al., 2009). Under abiotic stress, *AtGLR3.4* showed sensitivity to touch and cold (Meyerhoff et al., 2005). Alterations in pollen tube growth in *glr1.2* mutants (Michard et al., 2011) and root gravitropism in *glr3.3* mutants (Miller et al., 2010) might be related to impaired amino acid–gated Ca^2+^ signaling. Moreover, in *glr1.2* mutants, D-serine increased Ca^2+^ cytosolic concentration in the pollen tubes (Michard et al., 2011). Thus, the biological functions of GLRs in plants are not clear.

In plants, Ca^2+^ stimulates protein kinase activities through Ca^2+^-dependent protein kinases (CDPKs). The activities of CDPKs were first documented in pea (*Pisum sativum*) by Hetherington and Trewavas (1982). These unique enzymes are found in plants and some protozoa, and are characterized as Ca^2+^ sensors in plants. The CDPKs are encoded by a large multigene family consisting of 34 genes in *Arabidopsis* (*Arabidopsis* Genome Initiative, 2000) and 29 genes in rice (*Oryza sativa*). According to the conserved kinase domain sequence, CDPKs belong to a superfamily consisting of seven types of serine-threonine protein kinases, namely, CDPKs, CDPK-related kinases (CRKs), phosphoenolpyruvate carboxylase kinases (PPCKs), PEP carboxylase kinase-related kinases, calmodulin-dependent protein kinases, Ca^2+^ and calmodulin-dependent protein kinases, and sucrose nonfermenting-related serine/threonine-protein kinases (Hrabak et al., 2003). The protein sequences of CRKs are most similar to that of CDPKs.

The 14-3-3 proteins were initially discovered in mammalian brain tissues (Moore and Perez, 1967) and were named based on their chromatography elution and starch–gel electrophoresis profiles. In *Arabidopsis*, transcripts have been detected for 12 of the 15 14-3-3 genes (GF14/phi, GF14/chi, GF14/omega, GF14/psi, GF14/upsilon, GF14/lamda, GF14/nu, GF14/kappa, GF14/mu, GF14/epsilon, GF14/omicron, and GF14/iota) (Rosenquist et al., 2001; Sehnke et al., 2002). The 14-3-3 proteins can form either homodimers or heterodimers (Chang et al., 2009) and are scaffold proteins that can bind many proteins (Jaspert et al., 2011). In eukaryotes, 14-3-3 proteins regulate diverse cellular functions through hundreds of different protein–protein interactions. The biological functions of 14-3-3 proteins include kinase-mediated signal transduction, growth and development signaling, response to stress, and change of client protein activity (Kidou et al., 1993; Broadie et al., 1997; Camoni et al., 1998; Sehnke et al., 2002).

In plants, glutamate receptors are reported to be nonselective cation channels (NSCCs), which play a role in Ca^2+^ influx. Previous studies showed the biological functions of glutamate receptors in root elongation and abiotic stress responses. However, the functions of *GLRs* in plants are relatively unknown. Here, we identified and confirmed the phosphorylation site of AtGLR3.7, which can be phosphorylated by CDPK *in vitro*. The protein–protein interaction analyses carried out confirmed 14-3-3ω binding. We also investigated the biological function of *AtGLR3.7*. Results from the phenotyping of *glr3.7* mutants showed that *AtGLR3.7* is involved in salt stress response in *Arabidopsis*.

## Materials and Methods

### Plant Materials and Growth Conditions

All experiments were performed using *A. thaliana* Columbia ecotype (Col-0, wild type). Two *glr3.7* transfer DNA (T-DNA) insertion mutants, namely, *glr3.7-1* (SALK_022757) and *glr3.7-2* (SALK_103942), were ordered from ABRC (*Arabidopsis* Biological Research Center, http://abrc.osu.edu/), and *GLR3.7* overexpression (OE) lines (OE 5-6 and OE 10-2 and the serine point-mutated to alanine SA 10-2 and SA 15-6 lines) were generated by transgenics using the pEarleyGate104 vector. Seeds were surface sterilized with bleach [6% bleach: double distilled water (ddH_2_O) = 3: 7], and 0.02% Triton X-100 was applied for surface sterilization for 7 min followed by washes with sterilized ddH_2_O three times. Sterilized seeds were kept in darkness for 3 days at 4°C, then sown on solid half-strength Murashige and Skoog (MS) medium with 0.8% agar, and finally grown under long-day photoperiod conditions (16 h of light/8 h of darkness) at 22°C.

### Fusion Peptide Design and Construction

Forward and reverse primers around 50 to 60 nucleotides long were ordered to be self-ligated by temperature gradient and had sticky end of AscI and NotI restriction enzyme recognition site and APG amino acids after restriction enzyme recognition site. After phosphorylated by polynucleotide kinase at 5′ end for 30 min, the double strand primer was constructed into NRV vector harboring glutathione-S-transferase (GST), red fluorescence protein (RFP), and Strep-tags as previously described (Curran et al., 2011) (**Supplementary Table S1**) and transformed into BL21 for fusion peptide expression.

### Purification of GST-Tagged and 6His-Tagged Recombinant Proteins

*Escherichia coli* was incubated in the 40 mL 2xYT medium containing 200 μg ampicillin at 37°C overnight and then 400 mL 2xYT medium was added at 37°C for 3 h. After incubation, 220 μL isopropyl β-d-1-thiogalactopyranoside (IPTG, 1 M stock, 230 mg/ml) was added with final concentration of 0.5 mM and incubated 3 h at 28°C. The cells were centrifuged at 3,300 × *g* (Beckman Coulter J2-MC, USA) for 30 min under 4°C. The supernatant was discarded, and then 20 mL of lysis buffer containing 20 mM Tris pH 8.0, 500 mM NaCl, 10% glycerol, 0.5 mg/ml lysozyme, and 1 mM phenylmethylsulfonyl fluoride was added for 15 min. The cells were transferred to the 50 mL falcon tube and stored at −80°C. The cells were incubated in water around 40°C for dissolving, and the sonicator (Misonix XL2020, USA) was used to break the cells. After sonication, the cells lysate was centrifuged at 9,000 × *g* (Beckman Coulter J2-MC, USA) for 30 min under 4°C, and then supernatant was transferred to the new 50 mL falcon tube. One mL of GST-binding beads was added, which was washed with GST binding buffer containing 20 mM Tris pH 7.5, 100 mM NaCl, and 10% glycerol three times, and the solution was shaken for 1 to 3 h in the cold room. The GST binding beads were centrifuged, and the 10 mL Tris buffer (50 mM, pH7.5) was added to wash the beads, and the solution was transferred to the biospin columns (Bio-Rad); 1.5 mL 50 mM Tris buffer was used (pH 8.0), which contains 10 mM glutathione (30 mg/10 mL) to elute the recombinant proteins, and the elution was collected by a centrifugal filter (Amicon Ultra 30 K; Millipore). For 6His recombinant protein purification, *E. coli* was incubated in 40 mL 2xYT medium containing ampicillin (50 μg/mL) and shaking at 37°C overnight. Overnight culture was inoculated at 400 mL 2xYT with shaking at 37°C for 3 h and then induced with 0.5 mM IPTG and continue to grow at 28°C for 3 h. The cells were harvested by centrifugation at 9,000 × *g* (Beckman Coulter J2-MC, USA) for 30 min. The cell pellet was resuspended in lysis buffer and then stored at −80°C. The cells were thawed in 40°C water, sonicated, and then centrifuged for 30 min at 9,000 × *g* (Beckman Coulter J2-MC, USA). The supernatant was incubated with Ni-NTA resin (GE) at 4°C for 2 h and washed with first wash buffer containing 20 mM Tris pH 8.0, 10 mM imidazole, 500 mM NaCl, and 10% glycerol, and second wash buffer containing 20 mM Tris pH 8.0 and 100 mM NaCl. 6His recombinant proteins were eluted with 1.5 mL elution buffer containing 20 mM Tris pH 8.0, 300 mM imidazole, and 100 mM NaCl. The elution was collected with a centrifugal filter (Amicon Ultra 30 K; Millipore). Protein concentration measurement using Protein Assay Dye was based on the Bradford method (Cat. 500-0006; Bio-Rad).

### *In Vitro* Kinase Assay

It was reported that a synthetic peptide corresponding to the C-terminus of AtGLR3.7 was phosphorylated by CDPKs *in vitro* (Curran et al., 2011). In order to identify the phosphorylation site of AtGLR3.7, an *in vitro* kinase assay was carried out. According to the predicted conserved serine/threonine (Ser/Thr) residues, fusion peptides were designed for *in vitro* kinase assay (**Supplementary Table S1**). ACA2 and nitrate reductase can be phosphorylated by CDPKs (Douglas et al., 1998; Hwang et al., 2000), and its recombinant fusion peptides containing the phosphorylation sites were used as positive controls. Purification of GST-tagged kinases (GST-CDPK3-6H and GST-CDPK16-6H) and substrates (wild-type and mutant variants of GST-GFP-Strep-tagged fusion peptides, as shown in **Supplementary Table S1**), and the *in vitro* kinase assay were carried out as previously described (Curran et al., 2011). Cold ATP (50 μM, spiked with 1.25 μCi [γ-^32^P] ATP) was added to the reaction solution consisting of 30 ng purified CDPK, 3 μg fusion protein substrate, and 10 μL kinase reaction buffer, containing 20 mM Tris pH 7.5, 10 mM MgCl_2_, 1 mM EGTA pH 8.0, and 1.1 mM CaCl_2_, to start the kinase reaction. This reaction occurred at room temperature for 15 min, and it was stopped by adding 2 μL sodium dodecyl sulfate (SDS) sample buffer. All samples were loaded into a 12% SDS–polyacrylamide gel electrophoresis (PAGE) for electrophoresis, and γ-^32^P–labeled signals were normalized to the amount of protein, as determined from Coomassie brilliant blue stained gels after running SDS-PAGE. The γ-^32^P–labeled signals were detected on Typhoon 9400 image analyzer (GE Healthcare).

### Site-Directed Mutagenesis

Because the GLR3.7 fusion peptides contained three potential phosphorylation sites (Ser-859, Ser-860, and Thr-858), point mutations were created by site-directed mutagenesis of either Ser or Thr to alanine (Ala) to identify at which site the phosphorylation occurred (**Supplementary Table S1**). Site-directed mutagenesis was performed using the QuikChange Lightning kit (Stratagene). Two complementary oligonucleotides containing the desired mutation, flanked by unmodified nucleotide sequences, were designed. Mutated nucleotides were amplified by polymerase chain reaction (PCR), and 2 μL of *Dpn*I restriction enzyme was added directly to each amplification reaction and incubated at 37°C for 5 min to digest the parental supercoiled double stranded DNA (dsDNA). The *Dpn*I-treated DNA (2 μL) was then transformed into DH5α competent cells.

### Isolation of *Arabidopsis* Protoplasts

*Arabidopsis* protoplasts were isolated as previously described (Yoo et al., 2007). The leaves from 4-week-old plants were excised and treated with an enzyme solution (20 mM MES pH 5.7, 0.4 M mannitol, 20 mM KCl, 1% cellulose R10, 0.25% macerozyme R10, 20 mM KCl, 10 mM CaCl_2_, 5 mM β-ME, and 0.1% bovine serum albumin) at room temperature for 2 h. The enzyme solution containing *Arabidopsis* protoplasts was filtered through Miracloth and centrifuged in a 15-mL tube at 100 × *g* for 3 min (KUBOTA 2420) to pellet the protoplasts. The supernatant was removed, and the protoplasts were washed three times with W5 solution (154 mM NaCl, 125 mM CaCl_2_, 5 mM KCl, MES pH 5.7, and 5 mM glucose). The protoplasts were resuspended in MMG solution (MES pH 5.7, 0.4 M mannitol, and 15 mM MgCl_2_) to 2.5 × 10^5^ protoplasts per milliliter before p*olyethylene glycol* (PEG)–mediated transformation.

### Bimolecular Fluorescence Complementation

A previous study showed that GLR3.7 could be a client of 14-3-3ω in *Arabidopsis* (Chang et al., 2009). In bimolecular fluorescence complementation (BiFC) analysis, when two proteins interact with each other a yellow fluorescence signal is observed. Therefore, we used BiFC to confirm if GLR3.7 and 14-3-3ω have physical interaction. Because aminoacyclopropane-1-carboxylate synthase 7 (ACS7) and 14-3-3ω have been reported to interact in the cytosol (Yoon and Kieber, 2013; Huang et al., 2013), AtACS7 was used as the positive control. The BiFC analyses were carried out as previously described (Yoo et al., 2007; Liu et al., 2012). The open reading frame of *AtGLR3.7* was amplified from cDNA and then inserted into the pEarleyGate201-YN or pEarleyGate202-YC vector driven by the 35S promoter (from Keqiang Wu’s lab, National Taiwan University) that was fused to yellow fluorescent protein (YFP)-N or YFP-C, in frame. Plasmids (YFP-N and YFP-C; 10 μg) and protoplasts (200 μL) were transferred to a 15-mL round-bottom tube and gently mixed before the addition of 110 μL PEG solution followed by incubation at room temperature for 10 min. The PEG solution containing protoplasts was then diluted with 550 μL W5 solution and gently mixed. Protoplasts were centrifuged at 100 × *g* for 3 min (KUBOTA 2420) to pellet the protoplasts. The supernatant was removed, and the protoplasts were washed two times with W5 solution, resuspended in 1 mL W5 solution in each well of a six-well tissue culture plate, and incubated at room temperature. After 12 to 16 h, YFP fluorescence was detected by confocal microscopy (TCS SP5; Leica).

### Quartz Crystal Microbalance

The quartz crystal microbalance (QCM) analyses were carried out as previously described (Matsunaga and Ueda, 2010). Briefly, 500 μL of 1% SDS was added to the sensor, and after 3 min, ddH_2_O was used to wash the sensor. PIRANHA solution (99% H_2_SO_4_: 30% H_2_O_2_ = 3:1; 3 μL) was then added to the sensor, and after 5 min, ddH_2_O was used to wash the sensor. This procedure was repeated twice. The sensor was moved into the AFFINIX QN μ molecular interaction analyzer (Initium Inc.), and the basic frequency was measured. Phosphate-buffered saline (PBS; 500 μL) was added to the sensor, and after the frequency stabilized, the GST-GLR3.7 protein was added to the sensor until the sensor coating saturated. The sensor was washed twice with PBS, and 500 μL of this buffer was then added to the sensor. The 6His-14-3-3ω recombinant protein was added to the sensor, and the dissociation constant (Kd) value was measured using AQUA software (Initium Inc.).

### β-Glucuronidase Histochemical Analysis

For the β-glucuronidase (GUS) histochemical assay, the promoter region upstream of the start codon (2,000 bp) of *AtGLR3.7* was amplified and cloned into the binary vector pMDC163 using the Gateway cloning system (Thermo Fisher Scientific). The resulting construct (*proAtGLR3.7::GUS*) was transformed into *Arabidopsis* Col-0 plants *via* the *Agrobacterium tumefaciens* strain GV3101. The expression of GUS in the T3 transgenic lines was observed by immersing the seedlings in GUS staining buffer (0.2% Triton X-100, 1 mM potassium ferrocyanide, 1 mM potassium ferricyanide, and 100 mM NaPO_4_, pH 7) containing 1 mM 5-bromo-4-chloro-3-indolyl-b-d-glucuronide (X-Gluc) and placing them under vacuum for 10 min. After incubation at 37°C for 8 to 16 h, the staining buffer was removed, and samples were cleared by sequential 70% (v/v) ethanol immersion.

### Subcellular Localization of GLR3.7

The pEarleyGate 101 vector harboring 35S::GLR3.7-YFP was transformed into *Nicotiana benthamiana* by *A. tumefaciens* (GV3101) infiltration. Confocal microscopy (Delta vision, core; Applied Precision, Inc.) was used to observe the localization of the recombinant protein fused to YFP at the C-terminus of GLR3.7. AHA2 is a plasma membrane localization marker (DeWitt et al., 1996).

### RNA Extraction and PCR

Leaf samples were ground into powder with liquid nitrogen, and 1 mL REzolTM C&T reagent (PROTECH) with 200 μL of chloroform was used to isolate total RNA. Samples were centrifuged at 12,000 × *g* (Sigma 1-15K, Sigma Centrifuges) for 15 min and the resulting supernatants were moved to new 1.5-mL tubes. After adding 500 μL of isopropanol, samples were centrifuged at 12,000 × *g* (Sigma 1-15K) for 10 min. The resulting pellets were washed with 75% ethanol, resolved by diethyl pyrocarbonate (DEPC)-H_2_O, and 2 μg of each total RNA pellet were used for cDNA synthesis with the High-Capacity cDNA Reverse Transcription Kit (Applied Biosystems). The same amount of cDNA (2 μg) was used for semiquantitative PCR.

### Primary Root Phenotype

Seedlings were grown vertically on half-strength MS medium with 0.8% agar for 4 days. Four-day-old seedlings were transferred to half-strength MS medium containing 125 mM NaCl and grown vertically for 6 days (16 h of light/8 h of darkness). Primary root length was then measured using ImageJ software (http://rsbweb.nih.gov/ij/).

### Aequorin Bioluminescence

It is known that salt stress induces an increase in intracellular Ca^2+^ concentration, which in turn triggers a series of signal transmission reactions (Lynch and Lauchli, 1988; Lynch et al., 1989). Previous studies have also found that the cytosolic Ca^2+^ concentration was significantly lower in the *glr3.4* line than in Col-0 under salt-treated condition (Cheng et al., 2018). However, it is unclear whether GLR3.7 is involved in the regulation of cytosolic Ca^2+^ concentration. In the present study, 5-day-old seedlings were treated with 150 mM NaCl, and the aequorin (AEQ) luminescence test was used to determine Ca^2+^ concentration. *AEQ* Ca^2+^ reporter line was introduced for cytosolic Ca^2+^ concentration measurement (Singh et al., 2016). Seeds of cross lines (*AEQ* × *OE7-3*, *AEQ* × *glr3.7-1*, *AEQ* × *glr3.7-2*) were placed on half-strength MS medium and grown at long-day photoperiod condition (16 h light/8 h darkness) and 25°C for 5 days. On a 96-well plate, three 5-day-old seedlings per cell were soaked in 100 μL reconstitution buffer (2 mM MES [pH 5.7], 10 mM CaCl_2_, 10 μM coelenterazine [ALFA]) and cultured at 25°C in the dark, overnight. After two washes with coelenterazine-free buffer (2 mM MES [pH 5.7], 10 mM CaCl_2_), seedlings were soaked in 100 μL of coelenterazine-free buffer for 1 h and kept at 25°C in the dark to keep the seedlings in equilibrium for about 5 min. After removing 50 μL of the coelenterazine-free buffer solution, 50 μL (twice the final concentration of NaCl or glutamate) of treatment buffer (2 mM MES [pH 5.7], 10 mM CaCl_2_, 300 mM NaCl or 2 mM glutamate) was added. When the reaction terminated, 100 μL of discharge buffer (1 M CaCl_2_/20% ethanol) was added to react with the remaining AEQ. When the cold reading reached 1% of the highest value, the detection was stopped (Li et al., 2013). The luminescence measured 30 s before the salt treatment was used as the control value. Luminescence was measured in the Varioskan lux multimode microplate reader (Thermo Fisher Scientific), and all buffers were added to the specified well using an autoinjector. The instrument was set to detect luminescence every 1 s, and each detection time was 500 ms. The total detection time was 1 to 3 min. Relative luminescence units were transformed into cytosolic Ca^2+^ concentrations (pCa) based on: 0.332588 (−log k) + 5.5593, where *k* = is the luminescence at every second/total luminescence (Allen et al., 1977; Knight et al., 1996).

### Purification of Microsomal Fractions

*Arabidopsis* seedlings were suspension cultured in media containing 0.5 × MS medium supplemented with 0.5% sucrose, pH 5.7 at twilight (25°C/16-h photoperiod, 10 µE m-2 s-1 light) to enrich root growth for 2 weeks followed by 150 mM NaCl salt treatment for 3 days. Microsomal fractions were isolated as previously described (Hsu et al., 2009; Chang et al., 2012). Concentrations of membrane proteins were measured using the Bradford method.

### Western Blot

Anti-YFP antibody (catalog 66002-1-lg; Proteintech) (1:2,000) and anti–14-3-3 antibody (catalog AS12 2119; Agrisera) (1:2,000) were used to detect YFP and 14-3-3 proteins of membrane fractions. Polyvinylidene fluoride (PVDF) membrane (catalog 162-1077; Bio-Rad) was cut to fit the SDS-PAGE. The PVDF membrane was steeped in 100% methanol for 5 min. After composing the transfer sandwich cassette, transferring time was set for 1.5 h and current for 400 mA. Then 1× PBST buffer with 5% milk (Anchor, Taiwan) was used to blocking the PVDF membrane for 30 min. After blocking, the primary antibody was added to 10 mL 1× PBST with 5% milk (dilute 2,000 times) shaking overnight at 4°C. Then the membrane was washed by 1× PBST with 5% milk for three times. Secondary antibody was added to 10 mL 1× PBST with 5% milk (dilute 5,000 times) shaking 1.5 h at room temperature. Then the membrane was washed by 1× PBST with 5% milk for three times. Then 1× PBST without 5% milk was used to wash 5 min twice. The ECL reagents (Enhanced Luminol Reagent Plus, lot 275-081201; Oxidizing Reagent Plus, lot 265-081201; Blossom) was mixed 500 µL (1:1) well. The ECL mixed reagents were added into the membrane and detected by Luminescent image system (Wealtec KETAC, USA).

### Statistical Analyses

Each experiment was repeated at least three times. Values are expressed as the mean ± standard deviation in gene expression analysis and phenotypic analyses. Statistical analysis was carried out using Student *t* test and one-way analysis of variance (ANOVA) with *post hoc* Tukey honestly significant difference test.

## Results

### Ser-860 of GLR3.7 Is Phosphorylated by AtCDPKs *In Vitro*

**Figure 1:**
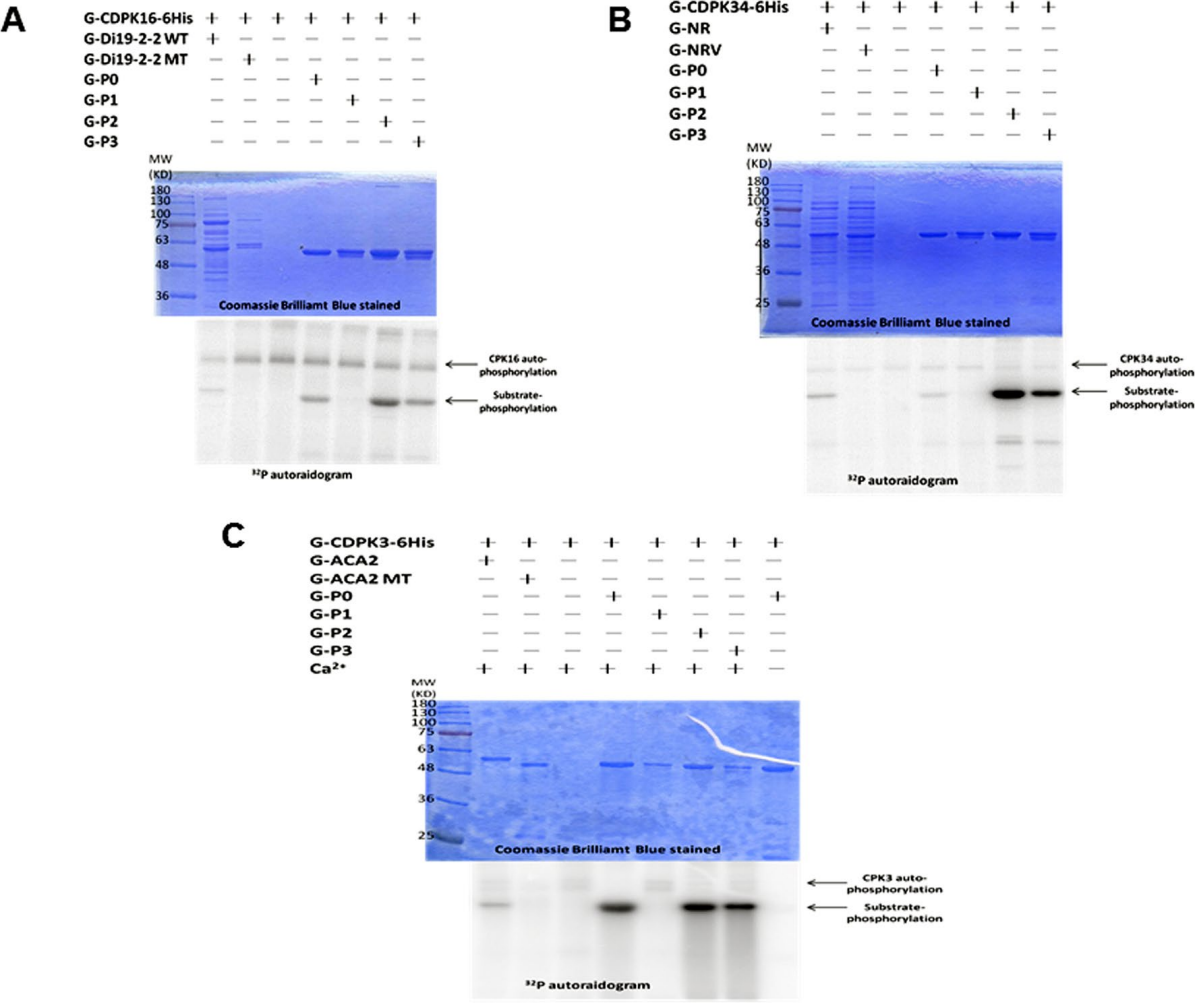
The site of AtGLR3.7 phosphorylated by AtCDPKs *in vitro*. Recombinant fusion peptides G-P0 (RYRRMERpTpSpSMPRA), G-P1 (RYRRMERpTpSAMPRA), G-P2 (RYRRMERpTApSMPRA), and G-P3 (RYRRMERApSpSMPRA) were used as substrates, and three recombinant kinases (G-AtCDPK16-6His, G-AtCDPK34-6His, and G-AtCDPK3-6His) were used to perform the kinase assay *in vitro*. The molecular weights of all fusion proteins were about 55 kD. The fusion peptide phosphorylation signal is marked with an arrowhead. The kinase autophosphorylation signal was about 100 kD. **(A)** The GLR3.7 fusion peptides were phosphorylated by G-AtCDPK16-6His *in vitro*. G-Di19-2-2 WT is also a fusion protein in which GST is fused with a peptide (DVLKSEQKEMpSYREDPY) recognized by G-AtCDPK16-6His; G-Di19-2-2 MT is similar to G-Di19-2-2 WT, but Ser was mutated to Ala. **(B)** G-NR is a fusion peptide in which GST is fused with a peptide of nitrate reductase (TLKRTApSTPFM) recognized by G-AtCDPK34-6His; G-NRV is a vector-only protein containing GST, RFP, and Strep-tags. **(C)** G-ACA2 is a fusion peptide in which the GST protein is fused with a peptide (RFRFTANLpSKRYEA) recognized by G-AtCDPK3-6His; G-ACA2 MT is similar to G-ACA2 but Ser was mutated to Ala. The results indicated that the fusion peptide of P1 could not be phosphorylated by CDPK, and Ser-860 of GLR3.7 is an *in vitro* CDPK phosphorylation site.

The fusion peptide containing the fragment of GLR3.7 P0 (RYRRMERTSSMPRA) was labeled by γ-^32^P in the autoradiogram (**Figure 1**). The results indicated that the GLR3.7 fusion peptide could be phosphorylated by recombinant G-CDPK16-6His (**Figure 1A**), G-CDPK34-6His (**Figure 1B**) and G-CDPK3-6His (**Figure 1C**) *in vitro*, individually. The kinase assay results indicated Ser-860 of AtGLR3.7 in the P1 fusion peptide as the phosphorylation site. The inactive kinase G-CDPK16-6His was introduced, and almost no phosphorylation of GLR3.7 P0 was detected, confirming that the phosphorylation of GLR3.7 P0 was due to CDPK16 activity (**Supplementary Figure S1**). In conclusion, Ser-860 of AtGLR3.7 was phosphorylated by CDPK3, CDPK16, and CDPK34 *in vitro*.

**Figure 2:**
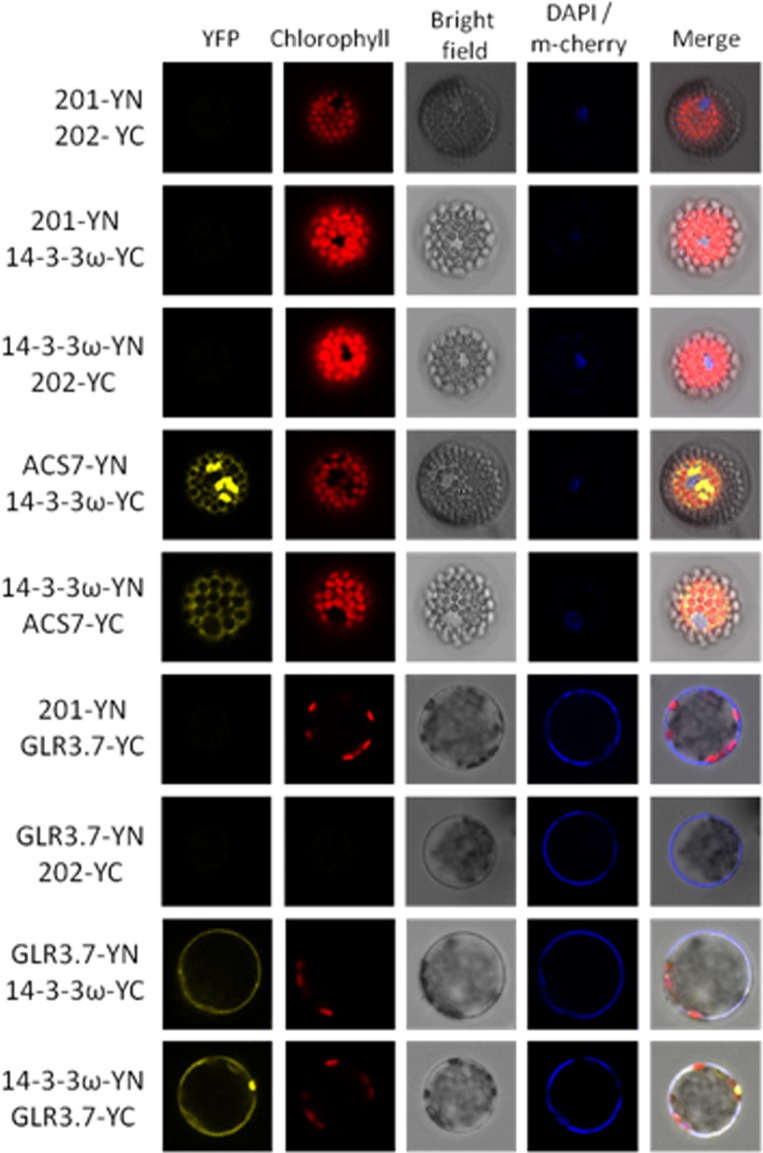
Protein–protein interaction between GLR3.7 and 14-3-3ω revealed by BiFC. Coexpression of cEYFP and nEYFP (negative control), nEYFP and 14-3-3ω-cEYFP (negative control), 14-3-3ω-nEYFP and cEYFP (negative control), 14-3-3ω-cEYFP and ACS7-nEYFP (positive control), 14-3-3ω-nEYFP and ACS7-cEYFP (positive control), nEYFP and GLR3.7-cEYFP (negative control), GLR3.7-nEYFP and cEYFP (negative control), GLR3.7-nEYFP and 14-3-3ω-cEYFP, GLR3.7-cEYFP and 14-3-3ω-nEYFP, and GLR3.7 and 14-3-3ω for testing interactions in *Arabidopsis* leaf protoplasts. The YFP signals were observed by confocal microscopy. DAPI was used to stain nuclei, and PIP2A-m-cherry to detect the plasma membrane. The blue signal indicates the nucleus stained with DAPI. The PIP2A-m-cherry is colocalized to the plasma membrane and also showed a blue signal. The red signal indicates chlorophyll autofluorescence; the YFP, DAPI, PIP2A-m-cherry, chlorophyll, and bright field signals are displayed. Based on transient expression results, GLR3.7 can physically interact with 14-3-3ω on the plasma membrane. ACS7 was used as the positive control for 14-3-3ω interaction (Huang et al., 2013).

### Protein–Protein Interaction Between GLR3.7 and 14-3-3ω Was Confirmed Using BiFC

**Figure 3:**
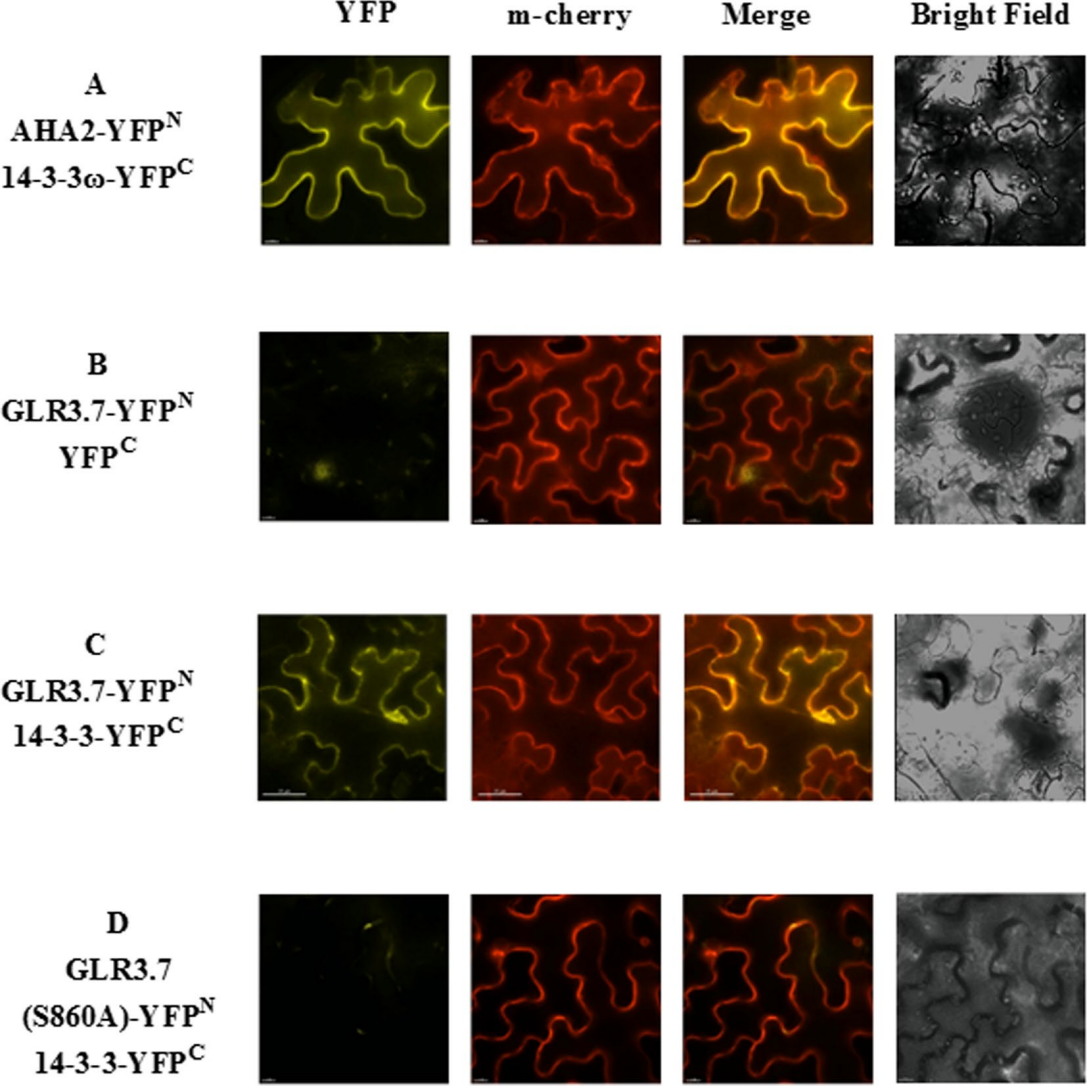
Physical interaction between GLR3.7 and 14-3-3ω in *Nicotiana benthamiana* in a transient expression assay. Protein–protein interaction was observed in the epidermal cells of *N. benthamiana*. Coexpression of **(A)** AHA2-YFP^N^ and 14-3-3ω-YFP^C^ (positive control), **(B)** GLR3.7-YFP^N^ and YFP^C^ (negative control), **(C)** GLR3.7-YFP^N^ and 14-3-3ω-YFP^C^, and **(D)** GLR3.7 S860A-YFP^N^ and 14-3-3ω-YFP^C^. The YFP signal was detected *via* the high-resolution live cell imaging system DeltaVision Core (Applied Precision, Inc.). The PIP2A-m-Cherry red fluorescent signal indicated plasma membrane. The Merge signal is the overlapping image of PIP2A-m-Cherry and YFP signals.

Our results indicated that GLR3.7 could physically interact with 14-3-3ω in the plasma membrane based on the results of the transient expression assay on *Arabidopsis* leaf protoplasts. In addition, using *A. tumefaciens*, we transiently expressed GLR3.7 in tobacco (*N. benthamiana*) epidermal cells and detected protein–protein interaction between GLR3.7 and 14-3-3ω by observing yellow fluorescent signals on the plasma membrane (**Figure 3**). AHA2 is a used as a positive control of 14-3-3 binding (Jahn et al., 1997). However, when Ser-860 was mutated to Ala, the yellow fluorescent signal on the plasma membrane disappeared (**Figure 3**), indicating that Ser-860 of GLR3.7 is the 14-3-3ω binding site.

### *In Vitro* 14-3-3ω Binding Assay Confirmed the Physical Interaction Between 6His-14-3-3ω and GST-GLR3.7

Most 14-3-3ω client interactions are thought to be promoted by phosphorylation of a target-binding site on the client. So far, three 14-3-3ω binding modes have been found: Mode-1 (K/R xx Sp/Tp *x* P) and Mode-2 (K/R_-4_-x_-3_-x_-2_-x_-1_-Sp/Tp-x_+1_-P_+2_), described by Muslin et al. (1996), and Mode-3 (Y_-1_TpV_+1_), described by Maudoux et al. (2000), where *x* represents any amino acid, and Sp/Tp is the phosphorylated site. Interestingly, the AtGLR3.7 phosphorylation site mapping indicated that Ser-860 fits into the conserved 14-3-3ω Mode-1, according to Scansite software (https://scansite4.mit.edu/4.0/#home) prediction. The Ser-860 of GLR3.7 had been pointed out as a 14-3-3ω binding site (Wudick et al., 2018), but it was predicted that it is the site for both CDPK phosphorylation and 14-3-3ω binding.

**Figure 4:**
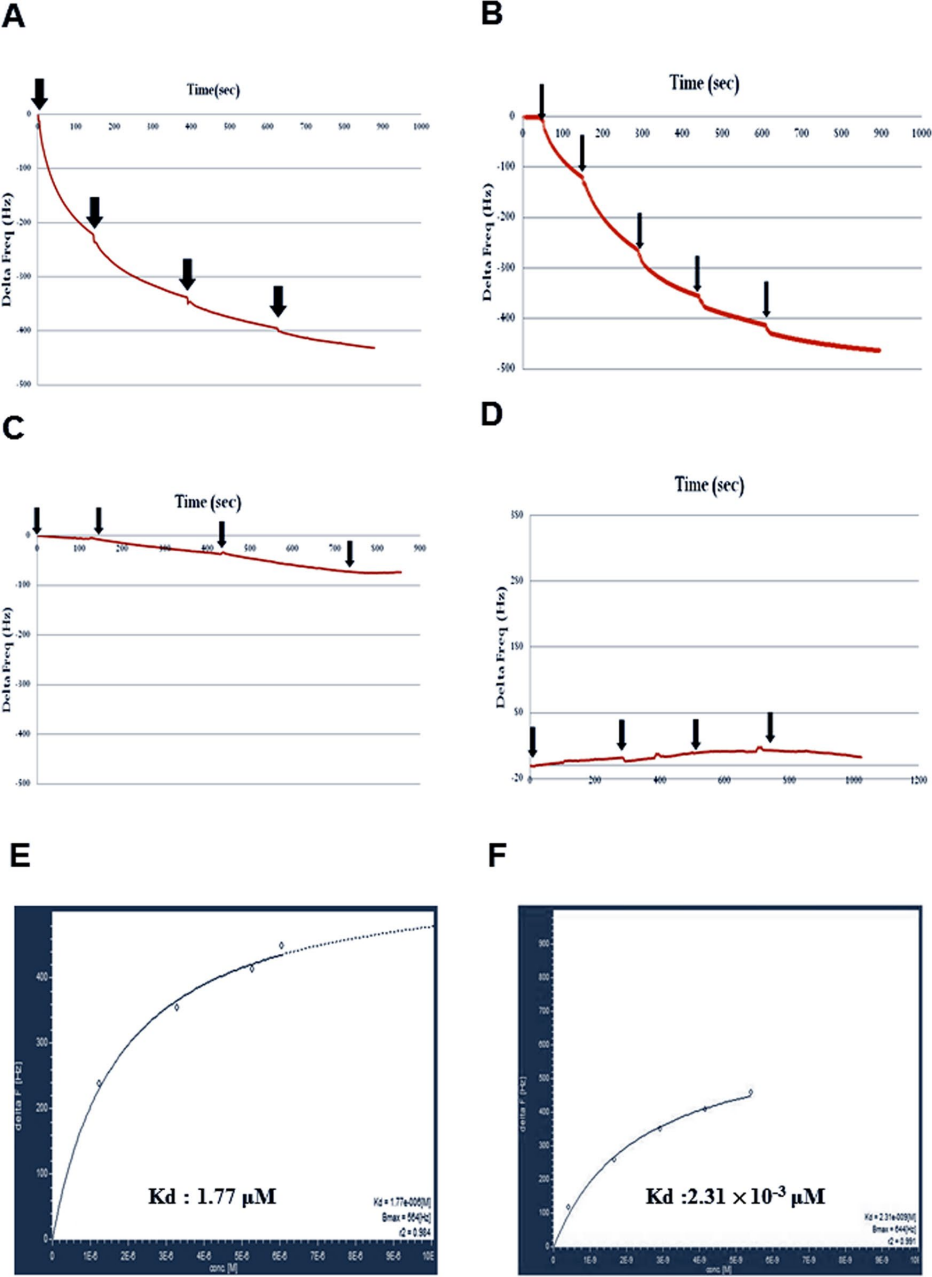
Protein–protein interaction between GST-GLR3.7 P0 and 6His-14-3-3ω revealed by using Quartz Crystal Microbalance. **(A)** G-P0 fusion peptide was coated on the sensor. **(B)** G-P0-phosphorylated fusion peptide was coated on the sensor. **(C)** G-P1 fusion peptide was coated on the sensor. **(D)** GST vector-only protein was coated on the sensor. The 6His-14-3-3ω protein was injected into the buffer separately (arrowhead). The frequency change was analyzed by AFFINIXQN software. Kinetic analysis of *in vitro* protein–protein interaction between recombinant protein GST-AtGLR3.7 P0 and 6His-14-3-3. QCM data of protein–protein interaction between 6His-14-3-3ω and GST-GLR3.7 P0 were analyzed by AQUA software, and Kd values were calculated. **(E)** The nonphosphorylated G-GLR3.7 P0 and 6his-14-3-3ω Kd were 1.77 × 10^−6^ M. **(F)** The phosphorylated G-GLR3.7 P0 and 6his-14-3-3ω were 2.31 × 10^−9^ M.

To further confirm whether Ser-860 of GLR3.7 is a 14-3-3ω binding site, the QCM analysis was carried out in an independent experiment. The recombinant fusion peptide GST-GLR3.7 P0 was coated on the sensor first, and then 6His-14-3-3ω protein was injected into the PBS buffer. If the recombinant peptide GST-GLR3.7 P0 could physically interact with 6His-14-3-3ω, the frequency of the sensor would change. Results showed that GST-GLR3.7 P0 interacted with 6His-14-3-3ω *in vitro* (**Figure 4A**), whereas GST-GLR3.7 P1 did not (**Figure 4C**). The GST vector was used as the negative control (**Figure 4D**). Our results supported the observation that Ser-860 is a 14-3-3ω binding site.

Quartz Crystal Microbalance data of protein–protein interaction between 6His-14-3-3ω and GST-GLR3.7 were further analyzed by AQUA software, which revealed a Kd value of 1.77 × 10^−6^ M (**Figure 4E**). A previous study showed that 14-3-3ω is a scaffold protein that can interact with clients that have already been phosphorylated (Muslin et al., 1996). Our results also showed that phosphorylated G-GLR3.7 (**Figure 4B**) could physically interact with 6His-14-3-3ω *in vitro* with a Kd as low as 2.31 × 10^−9^ M (**Figure 4F**).

### Promoter-GUS Assay

**Figure 5:**
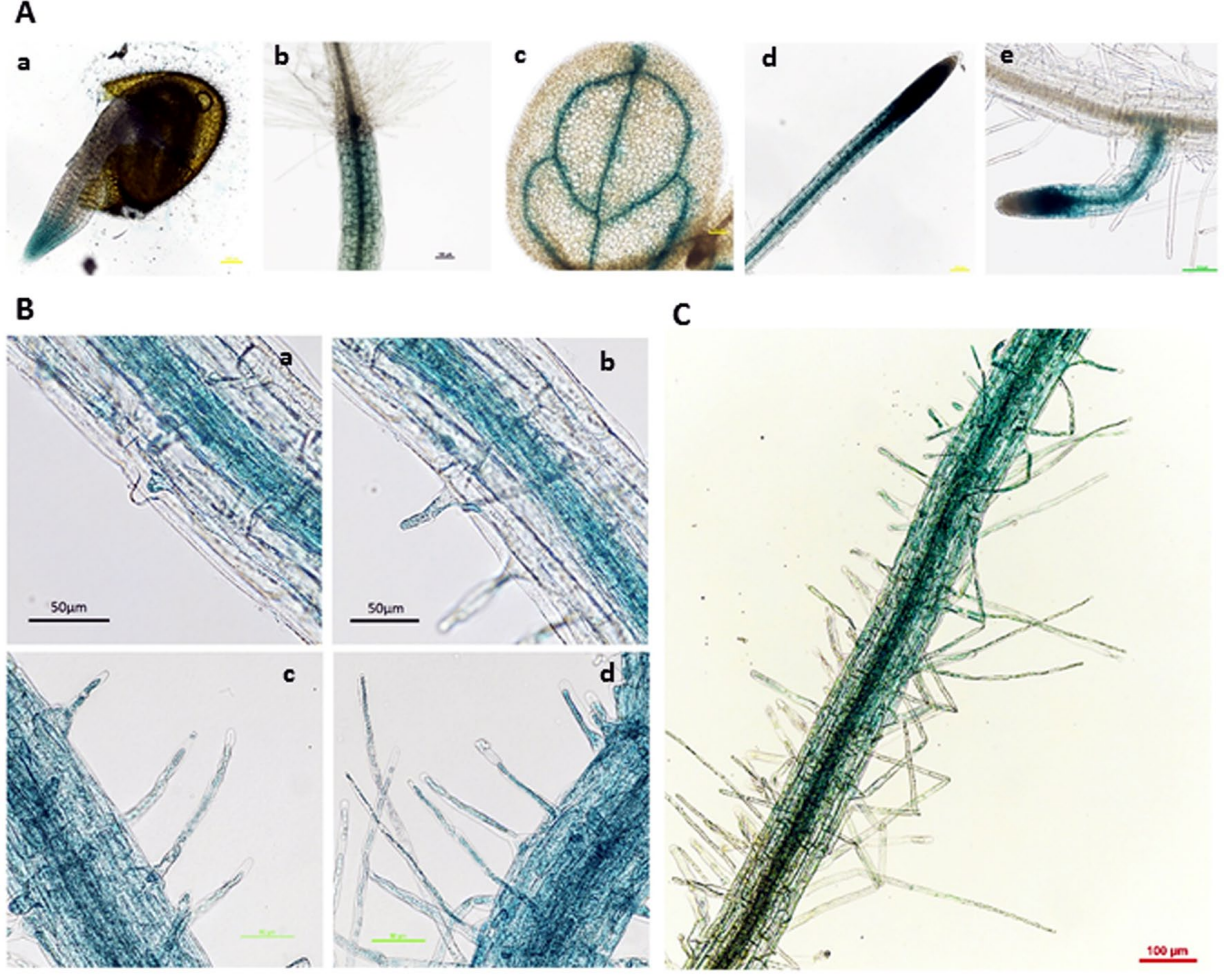
β-Glucuronidase (GUS) expression pattern of proGLR3.7::GUS in 3-day-old seedlings. **(A)** β-Glucuronidase expression in different tissues, including the radicle of 1-day-old seedlings (a), hypocotyl (b), leaf vein of 3-day-old seedlings (c), primary root (d), and lateral root of 5-day-old seedlings (e). Scale bars = 100 μm. **(B)** GUS expression in root hairs of the maturation zone near the root tip. Scale bars = 50 μm. **(C)** Root hairs on the maturation zone far from the root tip, where GUS expression was weak. Scale bars = 100 μm.

A previous study on transgenic plants harboring GUS driven by the *AtGLR3.7* native promoter, and examining a variety of different tissues and different stages of development, showed that *AtGLR3.7* is expressed in the cells of developing embryos, seedlings, and fully mature plants and flowers (Roy et al., 2008). In the present study, the GUS line driven by the 2-kb *AtGLR3.7* promoter displayed GUS activity in radicle, hypocotyl, vascular tissues of leaves, primary roots, and lateral roots (**Figures 5A, B**).

### AtGLR3.7 Is Localized to the Plasma Membrane

**Figure 6:**
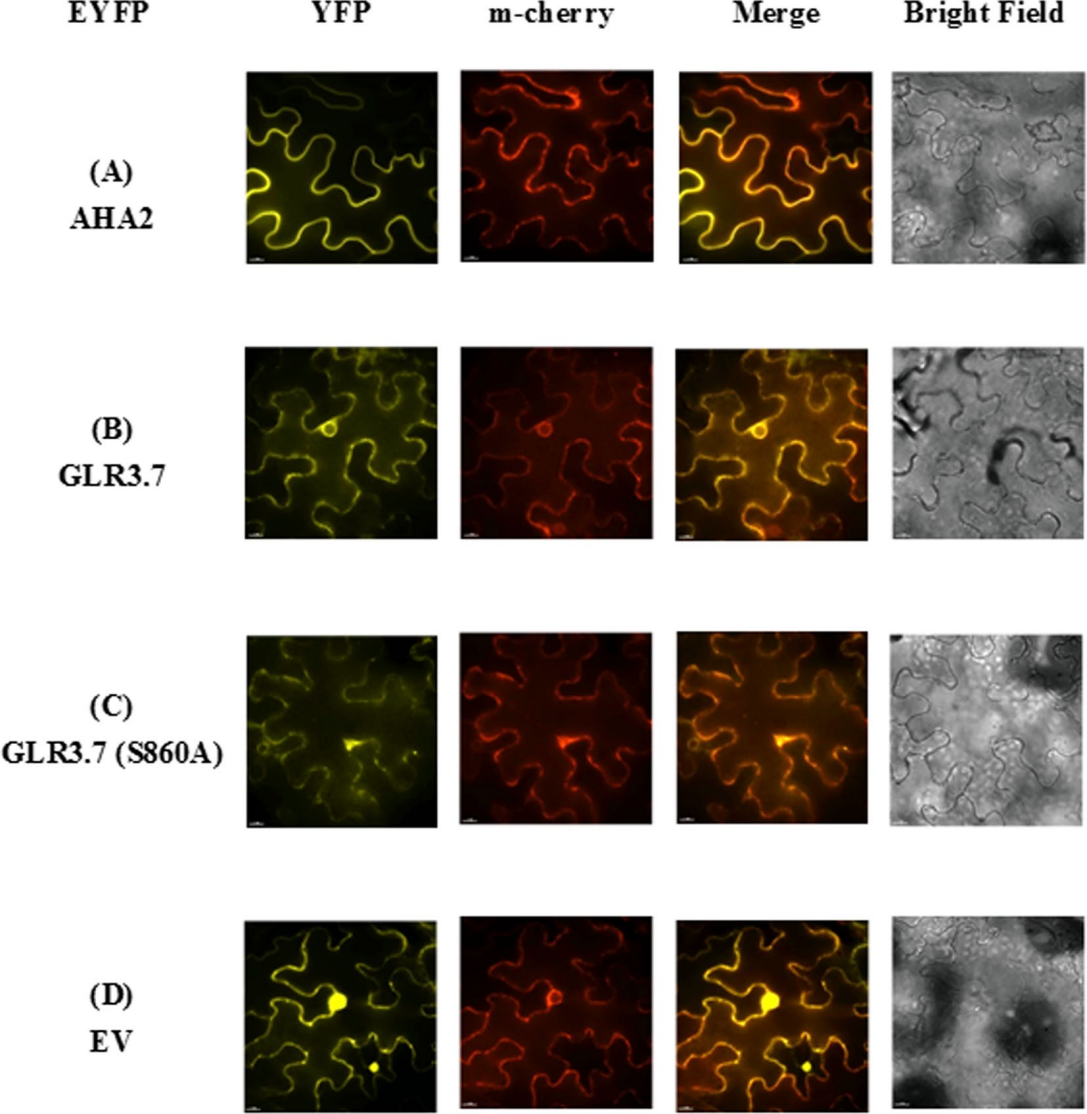
Subcellular localization of AtGLR3.7 in the epidermal cells of *Nicotiana benthamiana* in a transient expression assay. Expression of **(A)** AHA2-YFP (control), **(B)** GLR3.7-YFP, **(C)** GLR3.7 S860A-YFP, and **(D)** empty vector-YFP (negative control). The YFP signal was detected *via* the high-resolution live cell imaging system DeltaVision Core (Applied Precision, Inc.). The PIP2Am-Cherry red fluorescent signal indicates the plasma membrane. The Merge signal is the overlapping image of PIP2Am-Cherry and YFP signals.

The GLR proteins were found to be plasma membrane proteins (Lam et al., 1998). In order to further confirm whether AtGLR3.7 is actually localized to the plasma membrane, transient expression of GLR3.7 protein in *N. benthamiana* was carried out. The PIP2A-m-cherry was used as a plasma membrane marker (Nelson et al., 2007). As expected, the GLR3.7 protein is localized to the plasma membrane based on our transient assay, as its fluorescence signal overlaps with that of PIP2A (**Figure 6**).

### Seed Germination of *glr3.7-2* Is More Sensitive to Salt Stress

**Figure 7:**
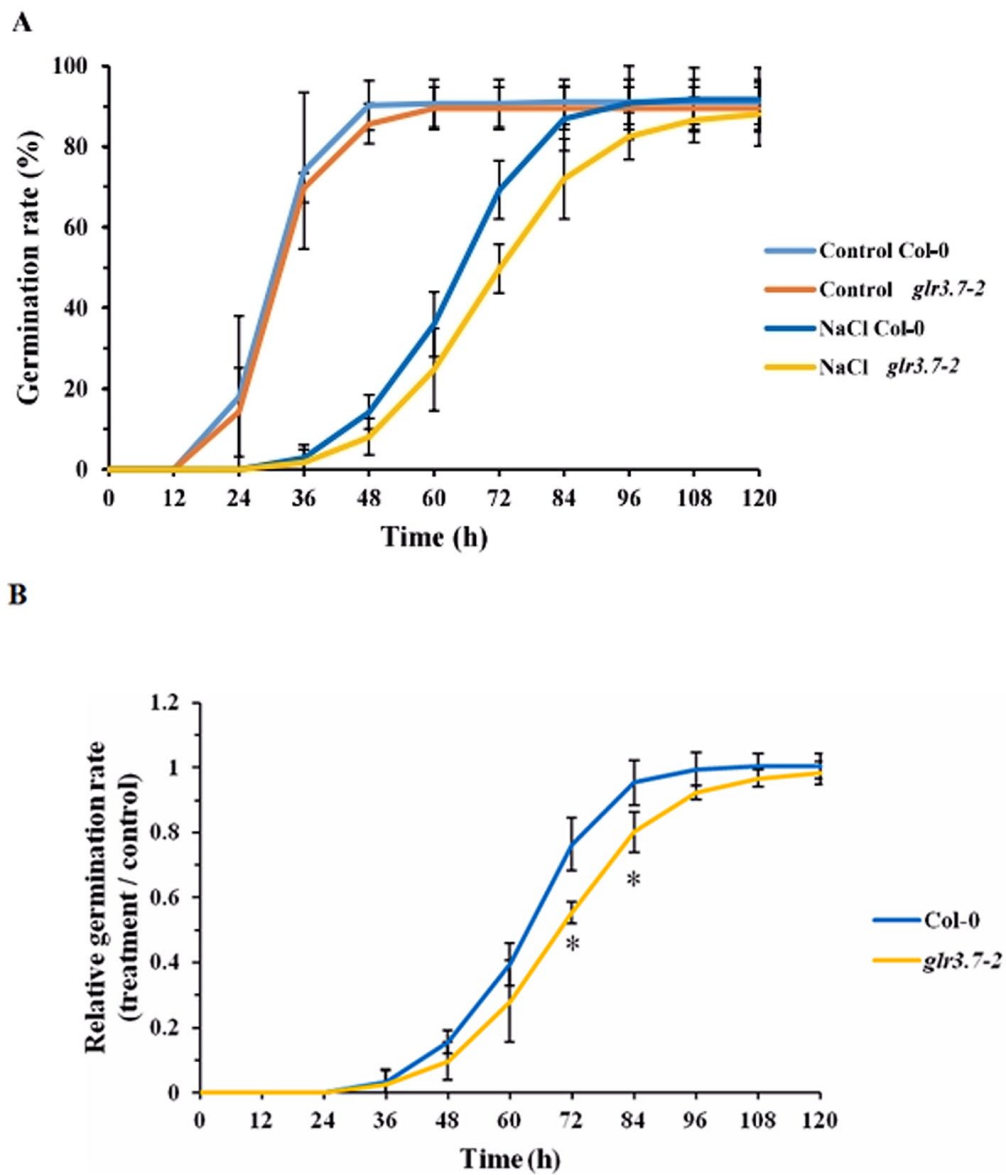
Seed germination of the *glr3.7-2* mutant line is more sensitive to salt stress than that of Col-0. Seeds of both lines were plated on half-strength MS medium containing H_2_O (control) or 125 mM NaCl for seed germination. Germination rate was observed every 12 h. **(A)** Seed germination rate. **(B)** Quantitative statistical analysis of seed germination rate: germination rate in the salt-treated group divided by the germination rate in the control group. The results were statistically analyzed by Student *t* test (mean ± SD, N = 3, total n ≥ 341, *P < 0.05).

To study the functions of *GLR3.7in planta*, two *glr3.7* T-DNA insertion mutants, *glr3.7-1* and *glr3.7-2*, were developed with T-DNA insertion sites at introns 1 and 2, respectively (**Supplemental Figure S2A**). The semiquantitative reverse transcriptase–PCR showed that *glr3.7-1* is a knockdown line, whereas *glr3.7-2* is a knockout line (**Supplemental Figures S2 B, C**). In a previous study, a *glr3.7* mutation line showed higher germination sensitivity under 150 mM NaCl treatment, by delaying germination time, than the wild type (Col-0) (Cheng et al., 2018). In the present study, we tested whether GLR3.7 is involved in seed germination under salt stress. Our results showed that *glr3.7-2* mutant strains significantly delayed seed germination at 72 and 84 h under salt stress condition in comparison to Col-0 (**Figure 7B**), which is consistent with the results from a previous study (Cheng et al., 2016). In addition, we also performed phenotyping of *cpk16-1* knockout mutant (SALK_020716) under salt stress condition. Treatment condition is the same as above. Results showed that *glr3.7-2* mutant strains significantly delayed seed germination at 72, 84, and 96 h under salt stress condition. These suggest that *CPK16* and *GLR3.7* are truly involved in salt stress response (**Supplementary Figures S3A, B**).

### Primary Root Growth of GLR3.7 Ser-860A Lines Is Less Sensitive to Salt Stress

**Figure 8:**
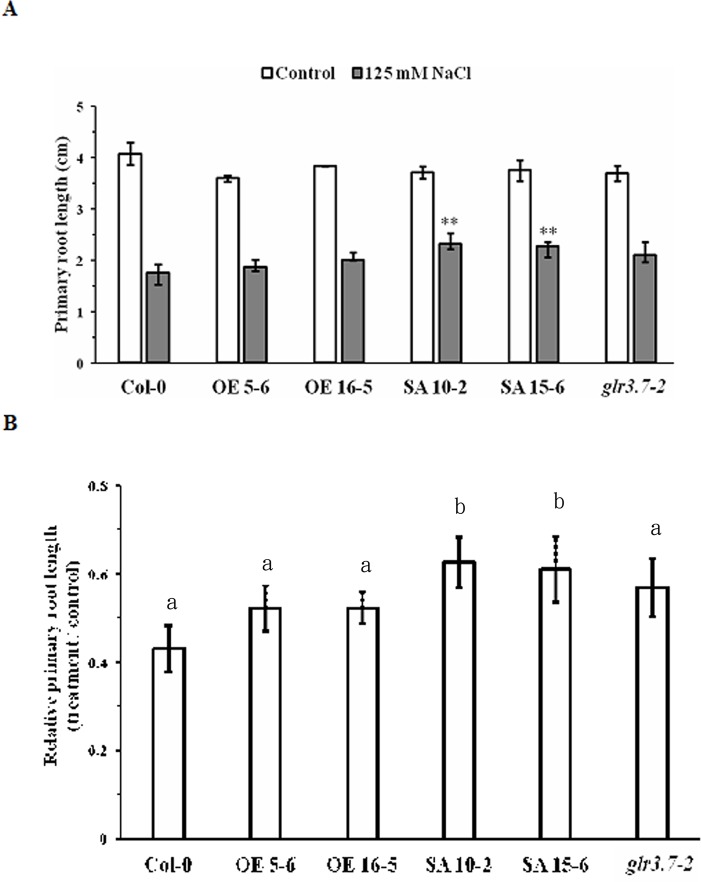
Primary root growth of GLR3.7-S860A overexpression lines is less sensitive to salt stress than that of Col-0. After 4 days of growth on half-strength MS medium, seeds of Col-0, *glr3.7-2*, GLR3.7 OE 5-6 and OE 16-5, and GLR3.7-S860A SA 10-2 and SA15-6 lines were transferred to half-strength MS medium containing H_2_O (control) or 125 mM NaCl. The seedlings were grown vertically for 6 days. **(A)** Primary root length of seedlings. **(B)** Quantitative statistical analysis of relative root length: root length in the salt-treated group divided by root length in the control group. The results of this experiment were statistically analyzed by Student’s *t*-test (mean ± SD, N ≥ 3, total n ≥ 26, *P < 0.05, **P < 0.01), and one-way ANOVA with *post hoc* Tukey honestly significant difference test (significantly at *P* < 0.05).

To test whether *GLR3.7* is involved in primary root growth under salt stress, seedlings were grown on half-strength MS medium for 4 days and then moved to half-strength MS medium containing H_2_O (control) or 125 mM NaCl, in which they were grown vertically for 6 days. Under the 125 mM salt stress condition, the primary root growth of the Ser-860A (Ser-860 point-mutated to Ala) overexpression lines SA 10-2 and SA 15-6 was less sensitive than that of other lines (**Figure 8B**). Relative primary root length (treatment/control) of SA 10-2 and SA 15-6 was around 60%, which was significantly higher than that of the *GLR3.7* overexpression lines OE 5-6 and OE 16-5 and wild type, suggesting that phosphorylation of Ser-860 may affect primary root growth under salt stress.

### Increase of Cytosolic Ca^2+^ Concentration by Salt Stress Is Significantly Lower in the *glr3.7-2* Mutant Line

**Figure 9:**
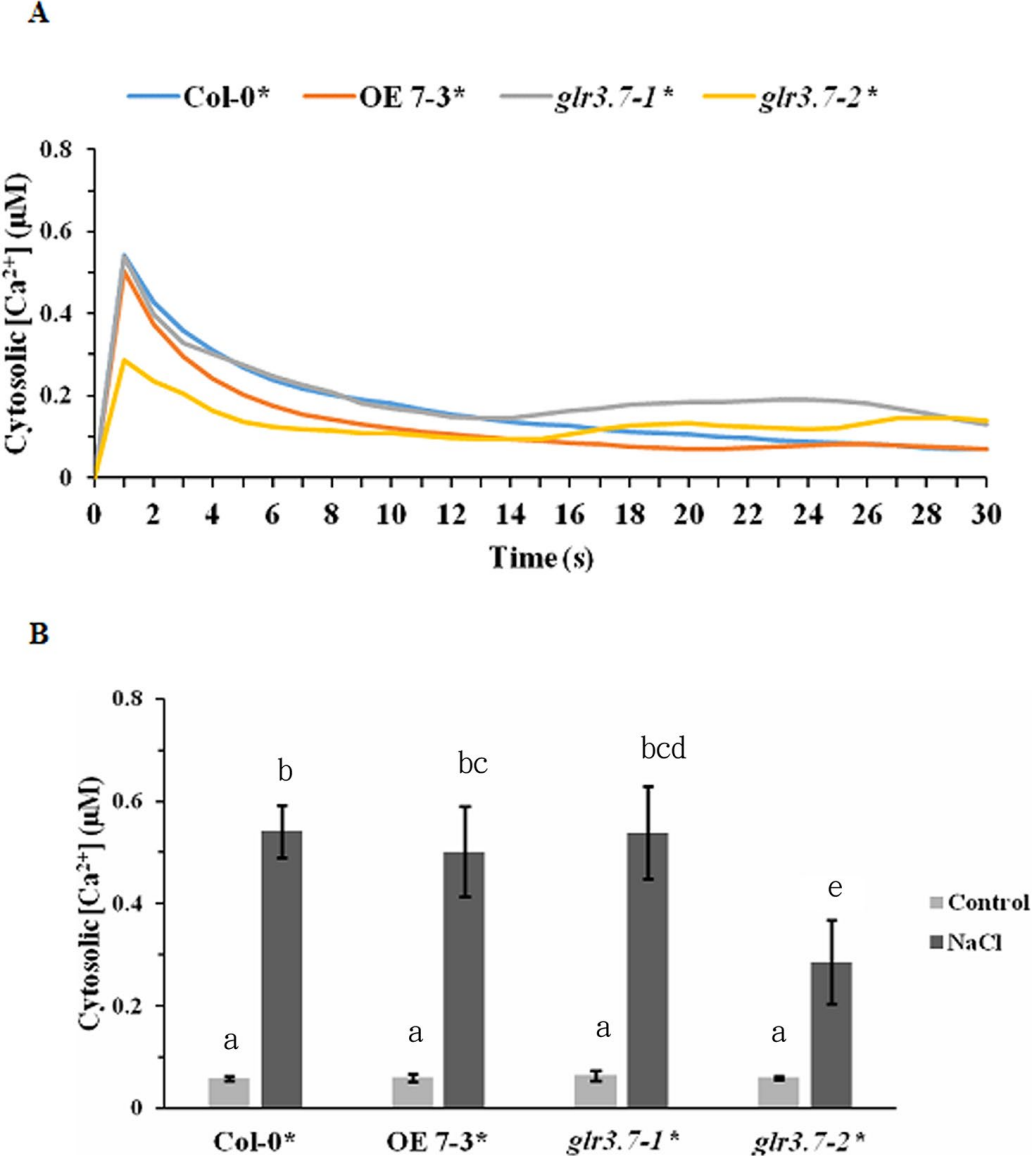
Increase of cytosolic calcium ion concentration by salt stress is lower in *glr3.7-2* mutant line than in Col-0. **(A)** Five-day-old seedlings of Col-0 aequorin transgenic line, *GLR3.7* OE line crossed to aequorin line, *glr3.7-1* mutant line crossed to aequorin line, and *glr3.7-2* mutant line crossed to aequorin line were subjected to 150 mM NaCl salt treatment. An aequorin bioluminescence assay was performed to measure the changes in cytosolic calcium ion concentration induced by the 150 mM NaCl treatment. **(B)** Quantitative analysis of cytosolic calcium ion concentration from the highest peak point in **(A)**. The control value corresponds to the cytosolic calcium ion concentration before NaCl treatment. The results of this experiment were statistically analyzed by one-way ANOVA with *post hoc* Tukey honestly significant difference test (mean ± SD, N = 3, total n = 24, significantly at *P* < 0.05).

Before the salt stress treatment, no significant increase was detected in the cytosolic Ca^2+^ concentration (**Supplemental Figures S4A–C**, **Figure 9**). However, after salt stress treatment, Col-0 showed a significant increase of the cytosolic Ca^2+^ concentration, but this increase was significantly lower in the mutant line *glr3.7-2* (**Figure 9B**). This indicated that *GLR3.7* is involved in the regulation of cytosolic Ca^2+^ concentration under salt stress.

### Association of 14-3-3 Proteins to the Microsomal Fractions Isolated From Salt-Treated GLR3.7-S860A Overexpression Lines Is Less Than GLR3.7 Overexpression Line

**Figure 10:**
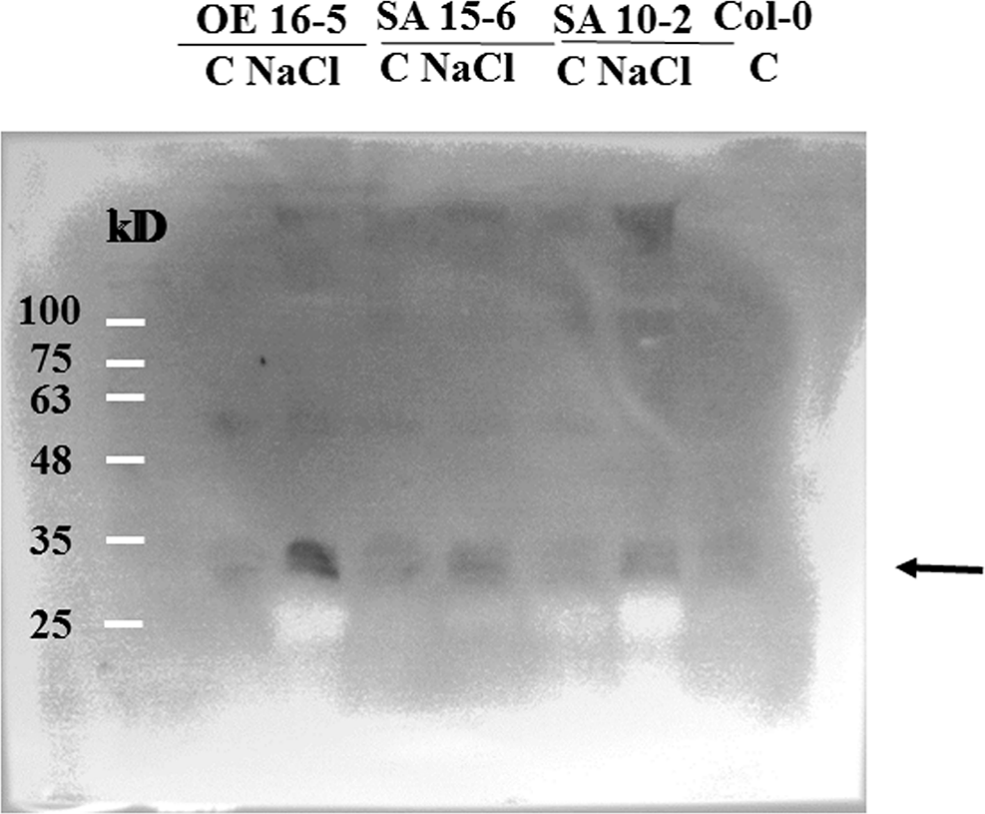
Western blot of association of 14-3-3 proteins to the microsomal fractions isolated from salt-treated GLR3.7-S860A overexpression line. C, control; NaCl, 150 mM NaCl salt treatment. Arrow indicates 14-3-3 isoforms detected by anti-14-3-3 antibody in Western blot. Col-0 was used as a control.

To determine whether association of 14-3-3 proteins to microsomal fractions in SA 10-2 and SA 15-6 is different from OE 5-6 and OE 16-5, microsomal fractions of 150 mM NaCl salt-treated-GLR3.7 overexpression lines, OE 5-6 and OE 16-5, and GLR3.7-S860A overexpression lines, SA 10-2 and SA 15-6, were isolated and subjected to Western blot. Results showed that under salt stress condition, association of 14-3-3 proteins to the microsomal fractions isolated from salt-treated GLR3.7-S860A overexpression lines SA 10-2 and SA 15-6 is less than GLR3.7 overexpression line OE 16-5 (**Figure 10**). This suggests that the phosphorylation of S860 is affected by salt stress.

## Discussion

Calcium, as a second messenger, is a ubiquitous signaling molecule in eukaryotic cells. The cytosolic Ca^2+^ signals that participate in nearly all aspects of plant growth and development encode information as binary switches or information-rich signatures (Spalding and Harper, 2011). Signaling occurs when the cell is stimulated to release stored Ca^2+^ or when Ca^2+^ enters the cell through the ion channels on the plasma membrane. In plants, Ca^2+^ levels can be regulated by Ca^2+^-binding proteins that function as Ca^2+^-signal sensors, which detect Ca^2+^ alterations by binding with domains such as EF hands (Dodd et al., 2010). These Ca^2+^ sensors include calmodulins, CDPKs, calcineurin B-like proteins (CBLs), and CBL-interacting protein kinases (Batistic and Kudla, 2004; DeFalco et al., 2009). A previous study showed that a fragment of AtGLR3.7 could be phosphorylated by CDPK *in vitro* (Curran et al., 2011). Accordingly, in the present study, we found that GST-GLR3.7 can be phosphorylated by recombinant CDPK16, CDPK34, and CDPK3 *in vitro* (**Figure 1**). In addition, our results showed that Ser-860 of AtGLR3.7 is a Ca^2+^-dependent phosphorylation site.

So far, three consensus phosphorylation motifs of CDPKs are known. The first consensus phosphorylation motif is ϕ_-5_-X_-4_-Basic_-3_-X_-2_-X_-1_-S, where the underlined S is phosphorylated, X is any residue, and ϕ is a hydrophobic residue (Bachmann et al., 1996; Huang and Huber, 2001; Harper and Harmon, 2005). The second consensus phosphorylation motif is Basic_-9_- Basic_-8_-X_-7_-Basic_-6_-ϕ_-5_-X_-4_-X_-3_-X_-2_-X_-1_-S-X_+1_-Basic_+2_. The third motif is ϕ_-3_-R_-2_-ϕ_-1_-S-ϕ_+1_-x- K_+3_-R_+4_ (Hernández-Sebastià et al., 2004). In the present study, the Ser-860 of AtGLR3.7 was phosphorylated according to the first CDPK phosphorylation motif. The phosphorylation site of GLR3.7 was not reported before, and it appears to be novel. In *Arabidopsis*, mutation of *CPK3* was found to affect seed germination rate under salt stress condition (Mehlmer et al., 2010). *cpk3* mutants exhibited salt-sensitive phenotype. In our study, *cpk16-1* knockout mutant lines showed salt stress-related phenotypes (**Supplementary Figure S3B**). Since GLR3.7 Ser-860A overexpression lines is less sensitive to salt stress in terms of primary root growth, the phosphorylation of AtGLR3.7 by CDPKs may be important in salt stress responses. However, whether AtGLR3.7 is a CDPK substrate, and Ser-860 is the phosphorylation site *in vivo* is critical. We have tried using membrane shaving followed by mass spectrometry analyses as previously described (Hsu et al., 2009), but did not detect phosphorylation site of AtGLR3.7 probably due to limitation of the technique. This requires further studies.

It has been reported that AtGLR3.7 may be a client of At14-3-3ω (Chang et al., 2009). The 14-3-3ω proteins are scaffold proteins that can interact with a phosphorylated protein and change the target protein activity (Sehnke et al., 2002). In animals, the activity of NMDA can be regulated by 14-3-3ω (Chen and Roche, 2009). The binding of 14-3-3ω proteins causes changes in client conformation, activity, localization, and association within larger protein complexes (Paul et al., 2012). However, there was no report on the interaction between At14-3-3ω and AtGLR3.7 in *Arabidopsis*, which was confirmed in the present study (**Figure 2**). We also found that CDPK phosphorylation and 14-3-3ω binding sites are overlapped, which is in agreement with CDPKs mediating 14-3-3ω binding in plants. For example, *Nicotiana tabacum* CDPK1 phosphorylation at Ser-114 represses shoot growth and promotes 14-3-3ω binding (Ormancey et al., 2017). In fact, 14-3-3 proteins were found to be associated with membrane proteins (Bunney et al., 2002). For example, 14-3-3 proteins bind the C-terminus phosphorylated Tyr residue of the plasma membrane H^+^–ATPase (Jahn et al., 1997; Fuglsang et al., 1999; Jelich-Ottmann et al., 2001). In addition, 14-3-3 proteins bind to potassium channel KAT1 in plants (Saponaro et al., 2017). In the present study, we found 14-3-3 association is less in AtGLR3.7 Ser-860A overexpression lines than GLR3.7 overexpression line under salt stress condition. This might indirectly explain the importance of AtGLR3.7 Ser-860 phosphorylation in 14-3-3 binding under salt stress condition.

Evidence is emerging for the NSCC function of AtGLR. The research about ion channel activity of glutamate receptors is still limited *in planta*. *Arabidopsis* plants overexpressing *AtGLR3.2* under the control of the 35S promoter showed symptoms of Ca^2+^ deficiency and hypersensitivity to potassium (K^+^) and sodium (Na^+^) ion concentrations (Kim et al., 2001). Antisense *AtGLR1.1* plants did not show hypersensitivity to K^+^ and Na^+^ compared with wild-type plants, but high levels of Ca^2+^ led to higher inhibition of their root growth (Kang and Turano, 2003). In *Arabidopsis* seedlings, AtGLR1.4 has been shown to function as a nonselective Ca^2+^-permeable cation channel and to mediate methionine-induced depolarization (Tapken et al., 2013). Altogether, these findings suggest that plant GLRs function in Ca^2+^ and monovalent cation transport and may form constitutively active ion channels. In *Xenopus* species oocytes, AtGLR3.7 has been successfully expressed and appears to function as a constitutively active channel catalyzing the voltage-independent movement of Na^+^, K^+^, and Ca^2+^ across the plasma membrane (Roy et al., 2008). Although we detected a lower increase of cytosolic Ca^2+^ concentration under salt stress (**Figure 9**) in *glr3.7* mutants than in Col-0, we did not assay channel activity changes for GLR3.7, which requires further study in the future.

In rat cerebellar granule cells, 14-3-3ω can interact with NMDA receptors and supports neuronal survival (Chen and Roche, 2009). Addition of 14-3-3ω proteins strongly increased the thiamin pyrophosphokinase 1 activity in a dose-dependent manner in *Arabidopsis* (Latz et al., 2007). Moreover, 14-3-3ω proteins are found to be involved in primary root growth regulation under abiotic stress conditions (van Kleeff et al., 2014). The quadruple mutant lines of *Arabidopsis* 14-3-3ω isoforms exhibited altered primary root growth under salt and mannitol treatments (van Kleeff et al., 2014). It was found that 14-3-3ωs play a positive role in primary root growth under normal condition, but a negative role under abiotic stresses (van Kleeff et al., 2014). In the present study, we found that the Ser-860 of GLR3.7 is a 14-3-3ω binding site. Mutation of this site to Ala affects primary root growth under salt stress. It is possible that the binding of 14-3-3ω inhibited primary root length under salt stress, thereby supporting the findings of van Kleeff et al. (2014). However, whether binding of 14-3-3ω affects the activity of AtGLR3.7 in *Arabidopsis* and whether AtGLR3.7 channel activity affects Ca^2+^ transport to regulate root growth requires further studies.

Glutamate receptors have been reported to be involved in seed germination in plants. In *Arabidopsis*, mutation of *GLR3.4* led to more sensitivity to ABA and affected seed germination (Cheng et al., 2018). Repression of *GLR3.5* led to higher sensitivity to ABA and delayed seed germination, whereas overexpression of *GLR3.5* led to reduced sensitivity to ABA and earlier seed germination (Kong et al., 2015). Our results showed that *GLR3.7* could also be involved in the regulation of seed germination (**Figure 7**), although this might be ABA-dependent or not. In *glr3.4* mutants, cytosolic Ca^2+^ concentration increased less than in wild-type seedlings under salt stress (**Figure 9**), consistent with that previously reported by Cheng et al. (2018).

Glutamate receptors were found to be involved in root growth and development. In *Arabidopsis*, *GLR3.6* regulates primary and lateral root development through regulating the cell cycle control gene *KRP4*, a Kip-related protein (Singh et al., 2016), and GLR3.2 and GLR3.4 may form a protein complex that regulates lateral root initiation (Vincill et al., 2013). In rice, mutation of *GLR3.1* led to short-root phenotype (Li et al., 2006). However, these effects were found under normal conditions. In the present study, we observed changes in the root phenotype of the *GLR3.7* (S-860A) overexpression line under salt stress (**Figure 8**). Because GLR3.7 diverged evolutionarily from other Clade III members (Chiu et al., 2002), it might have specialized in regulating primary root growth under abiotic stress conditions.

## Data Availability Statement

All datasets for this study are included in the manuscript/**Supplementary Files**.

## Author Contributions

P-HW conducted the kinase assay and QCM analysis. C-EL performed BiFC analysis, subcellular localization analysis, AEQ luminescence assay, and phenotyping of mutant lines. Y-SL isolated T-DNA insertion lines and generated *AEQ* cross lines. M-HL isolated membrane fractions for Western blot. P-YC conducted phenotyping of *cpk16* mutant lines. H-CC participated in the kinase assay. I-FC led the whole project including experimental designs.

## Funding

The present study was supported by the Ministry of Education, Taiwan, through the National Taiwan University, Taiwan (grants 107L893106, and 108L893106) and by the Ministry of Science and Technology, Taiwan (grants MOST #104-2311-B-002-034, 104-2311-B-002-005, 105-2313-B-002-006, 106-2313-B-002-014, 107-2313-B-002-002, 108-2311-B-002-002, and 108-2313-B-002-048), through the funds attributed to I-FC.

## Conflict of Interest

The authors declare that the research was conducted in the absence of any commercial or financial relationships that could be construed as a potential conflict of interest.
