# Supplementary material for: The Glutamate Receptor-Like Protein GLR3.7 Interacts With 14-3-3ω and Participates in Salt Stress Response in Arabidopsis thaliana
Source: Front Plant Sci. 2019 Sep 30;10:1169. doi: 10.3389/fpls.2019.01169 (PMC6779109; doi:10.3389/fpls.2019.01169)

**Supplemental files**

**Supplementary Table S1. Fusion peptides used in the in vitro kinase assay**

| Name | Amino acid sequence |
| --- | --- |
| Di19-2-2 WT | DVLKSEQKEMpSYREDPY |
| Di19-2-2 MT | DVLKSEQKEMAYREDPY |
| NR | TLKRTApSTPFM |
| ACA2 | RFRFTANLpSKRYEA |
| ACA2 MT | RFRFTANLAKRYEA |
| GLR3.7P0 | RYRRMERpTpSpSMPRA |
| GLR3.7 P1 | RYRRMERpTpSAMPRA |
| GLR3.7 P2 | RYRRMERpTApSMPRA |
| GLR3.7 P3 | RYRRMERApSpSMPRA |
| GLR3.6 P4 | EGpSIRRRpSpSPpSA |
| GLR3.6 P5 | EGpSIRRRApSPpSA |
| GLR3.6 P6 | EGpSIRRRpSAPpSA |
| GLR3.6 P7 | EGpSIRRRpSpSPAA |
| GLR3.6 P8 | EGAIRRRpSpSPpSA |
| GLR3.6 P9 | EGAIRRRAAPAA |
| GLR3.6 P10 | EGSIRRRAAPSA |
| GLR3.6 P11 | CPEEAEGpSIRRR |
| GLR3.6 P12 | CPEEAEGAIRRR |
| GLR3.6 P13 | PEEAEGpSIRRRpSpSP |

A. pS representsphosphor-serine

B. Gray bars represent point mutated amino acid residue by site-directed mutagenesis

**Supplementary Fig. S1. Phosphorylation of GLR3.7 by CDPK16 *in vitro.*** Recombinant fusion peptides GLR3.7-P0 (RYRRMERTSSMPRA) was used as substrates, and recombinant kinase (AtCDPK16-6His) was used to perform the kinase assay *in vitro*.


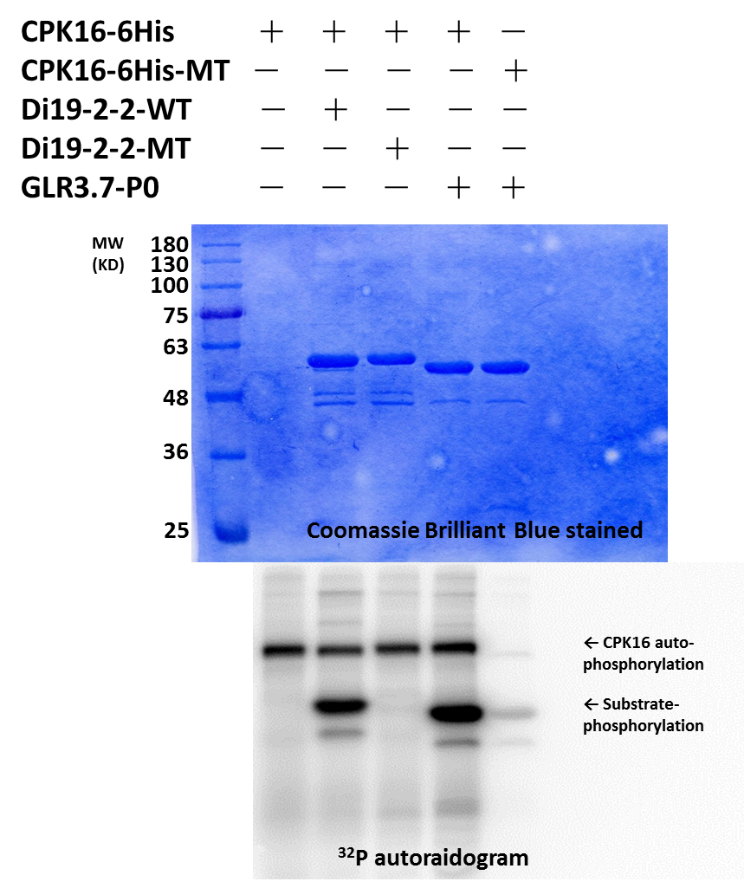


**Preparation of *E. coli* DH5α competent cells**

Single colony of *E. coli* DH5α competent cell was inoculated into 5 ml LB medium (1 % trypton, 0.5 % yeast extract, 1 % NaCl) with shaking at 37°C for 16 hours, then added the cells to the 1 liter flask containing 500 ml SOB medium with shaking at 37°C for 3 hours. The cells were centrifuged at 2700 x g (Beckman Coulter J2-MC, USA) at 4°C for 10 min, and resuspended gently in 130 ml 0.1 M CaCl_2_ solution. Then the cells were centrifuged at 2700 x g at 4°C for 10 min and the supernatant was removed. Finally cells were resuspended in 4 ml TB buffer and separated into 1.5 ml eppendorf tube.

***E. coli* transformation**

DH5α competent cells were put on ice, and 1 μl plasmid was added to thaw cells. The cells were placed on ice for 30 min and heat-shocked for 90 seconds in 42°C, then the cells were put on ice for 2 min. One ml LB medium was added to let the cells recovered at 37°C for 30 min. After recovery, the cells were spread on plates and incubated at 37°C overnight.

**Supplementary Fig. S2. Genotype and transcription analysis of *glr3.7* T-DNA insertion lines.** (A) The T-DNA insertion site for *glr3.7-1* mutant line (SALK_022757) is at intron 1. The T-DNA insertion site for *glr3.7-2* mutant line (SALK_103942) is at intron 2. (B) The mutant line was isolated as a homozygous line through PCR using the primers (LBb1.3, LP, RP) suggested at the SIGnAL website. (C) GLR3.7 gene expression level showed the *glr3.7-1* mutant line is a knockdown line and *glr3.7-2* mutant line is a knockout line. *UBQ10* is an internal control.

**Supplementary Fig. S3.** **Seed germination of the cpk16-1 mutant line is more sensitive to salt stress than that of Col-0.** Seeds of both lines were plated on half-strength MS medium containing H_2_O (control) or 125 mM NaCl for seed germination. Germination rate was observed every 12 h. **(A)** Seed germination rate. **(B)** Quantitative statistical analysis of seed germination rate: germination rate in the salt-treated group divided by the germination rate in the control group. The results were statistically analyzed by Student’s *t*-test (mean ± SD, N = 3, total n > 300, * p < 0.05).


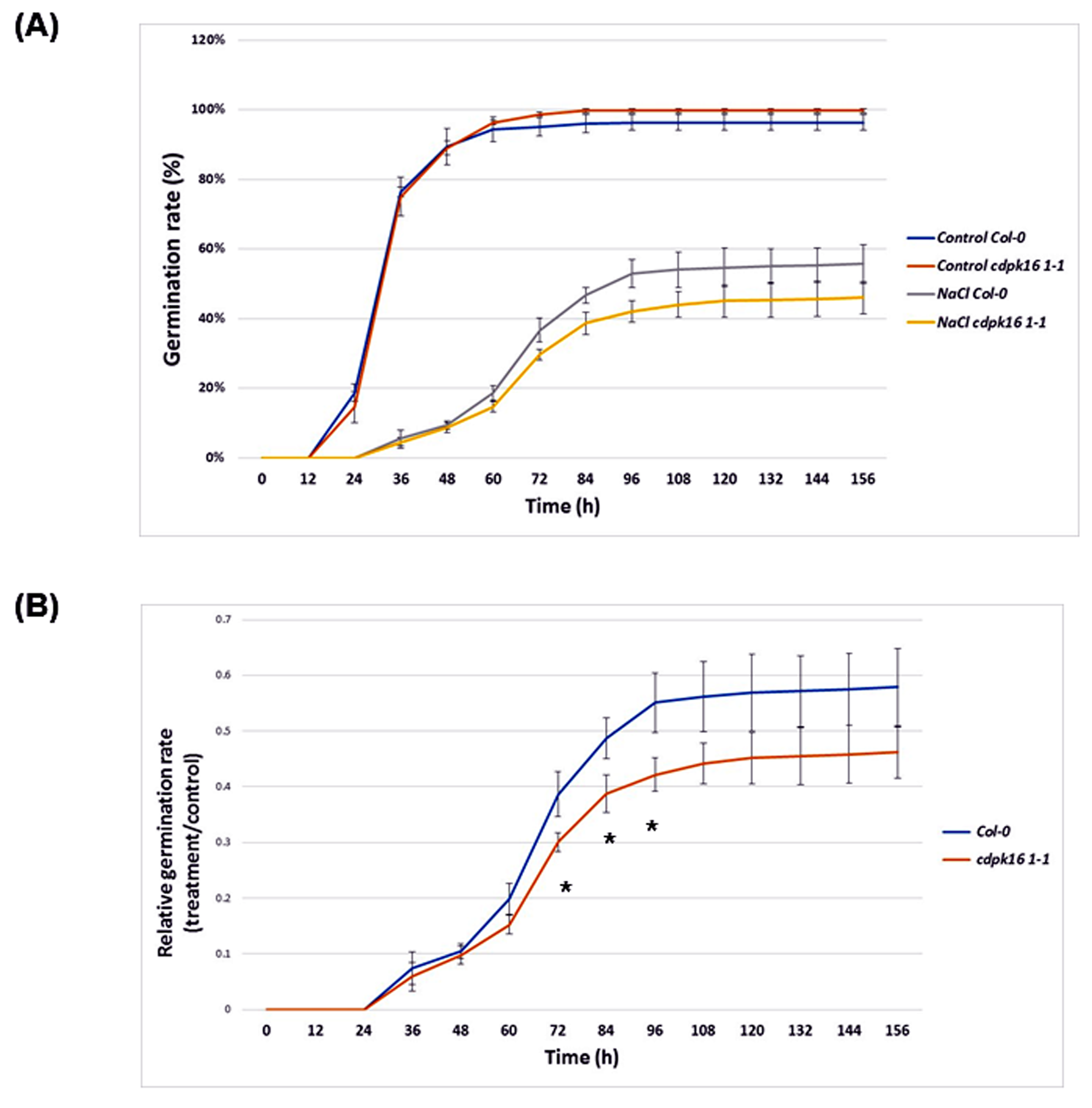


**Supplementary Fig. S4.** **Cytosolic calcium ion concentration under normal and salt stress conditions.** Five-day-old seedlings of Col-0 Aequorin transgenic line, *GLR3.7* OE line crossed to Aequorin line, *glr3.7-1* mutant line crossed to Aequorin line, and *glr3.7-2* mutant line crossed to Aequorin line were subjected to 150 mM NaCl salt treatment followed by Aequorin bioluminescence assay to measure cytosolic calcium ion. Cytoplasmic calcium ion concentration was all induced by 150 mM NaCl (A~C). Control shows cytoplasmic calcium ion concentration before NaCl treatment (D~F).

**(A)**

**
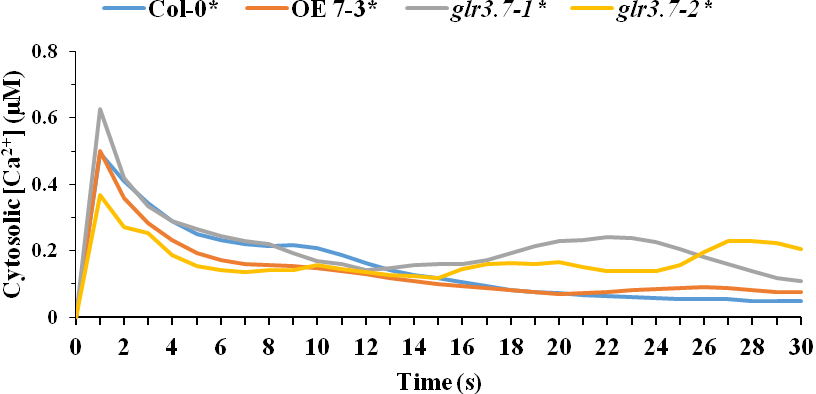
**

**(B)**

**
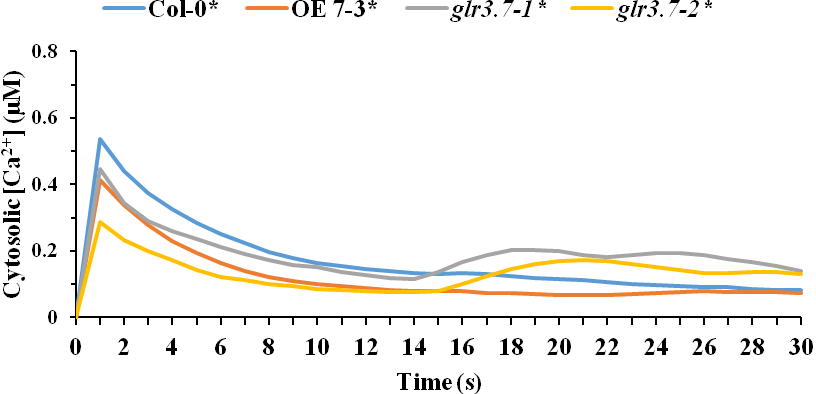
**

**(C)**


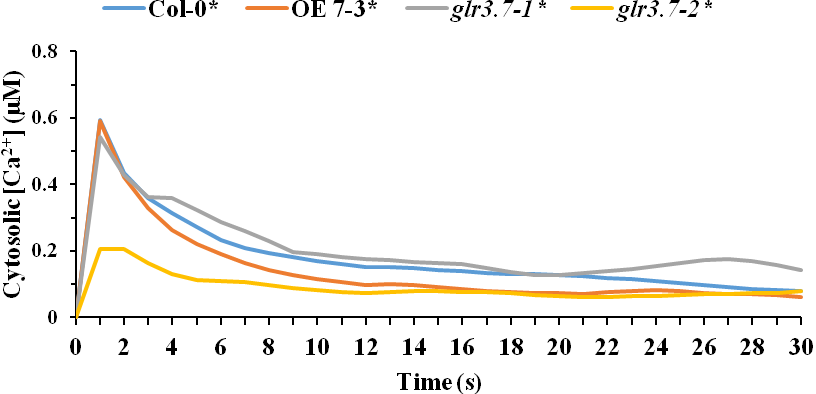


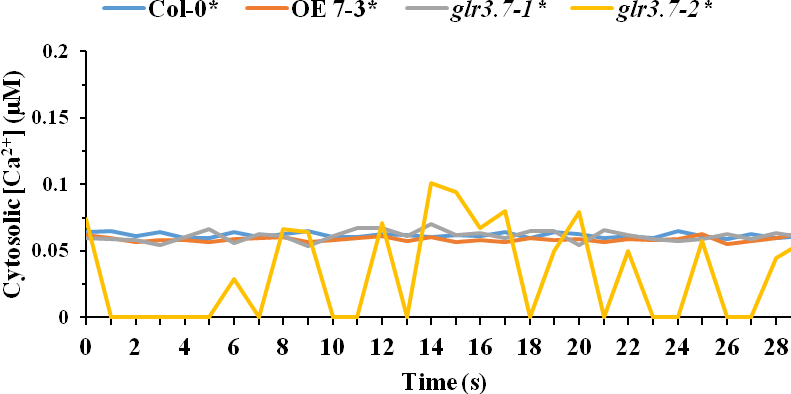


**(D)**

**(E)**


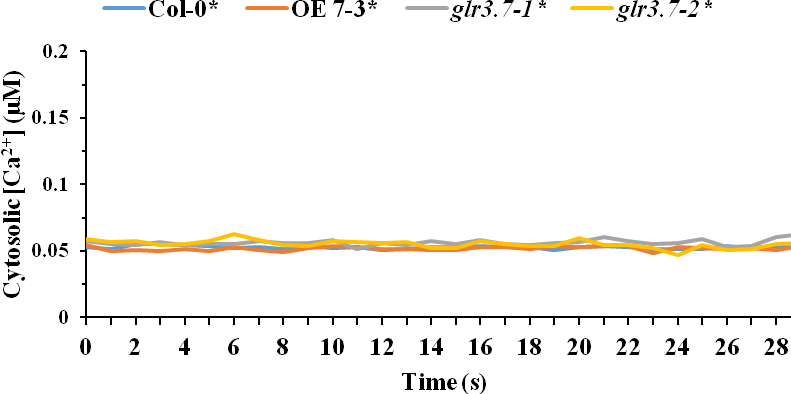


**(F)**


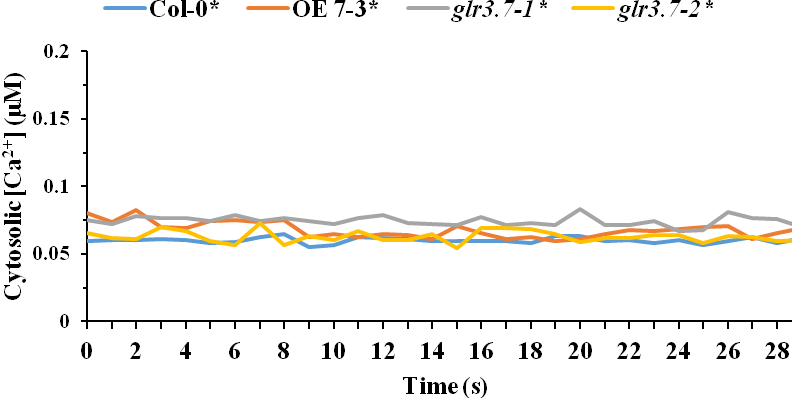

Supplement: Supplementary file 1 [file Table_1.doc]
